# Clinical Presentation of Usher Syndrome Type 1B (USH1B) in a 10-Month-Old: A Case Report

**DOI:** 10.7759/cureus.43934

**Published:** 2023-08-22

**Authors:** Meghan J Filson, Dakota C Davis, Claire Yother

**Affiliations:** 1 Medicine, Edward Via College of Osteopathic Medicine, Auburn, USA; 2 Medicine, Alabama College of Osteopathic Medicine, Dothan, USA; 3 Pediatrics, Gadsden Pediatric Clinic, P.A., Gadsden, USA

**Keywords:** medicine-pediatrics, cochlear implant (ci) surgery, universal newborn screening, deaf-blindness, usher syndrome, pediatric genetics, vestibular dysfunction, autosomal recessive retinitis pigmentosa, congenital sensorineural hearing loss

## Abstract

*Usher Syndrome (USH)* is a genetically inherited condition characterized by congenital sensorineural hearing loss and progressive vision loss secondary to retinitis pigmentosa. Patients may also display vestibular areflexia and balance issues secondary to inner ear damage. Usher Syndrome is the most commonly diagnosed syndrome within the blind-deaf community, and it accounts for a significant portion of the hearing and visual deficit cases among patients younger than 65 years of age. Due to the reported prevalence of Usher Syndrome in the United States, it appears there is chronic underdiagnosis in clinical settings throughout the country. A possible explanation for this is the visual deficits of Usher syndrome do not appear until later in life and thus inappropriately lower the index of suspicion for this diagnosis in young children with hearing deficits. This case study highlights a healthy newborn who failed the universal newborn hearing screening (UNHS) bilaterally and a follow-up hearing screening in a pediatrician's office. Auditory brainstem response (ABR) later confirmed bilateral severe-to-profound sensorineural hearing loss. Upon genetic testing, an abnormality in the Unconventional Myosin VII-A (MYO7) gene was discovered and consistent with Usher syndrome Type 1B (USH1B). Usher Syndrome should be considered on the differential for patients with congenital hearing loss. Genetic counseling should be used if no other cause of sensorineural hearing loss is identified. Due to the progressive nature of this condition and the physical and developmental deficits that will transpire without treatment, a genetic panel for hearing loss should be prioritized to determine the presence of genetic mutations suggesting Usher syndrome.

## Introduction

Usher Syndrome (USH) is the most common cause of syndromic hearing loss after Pendred syndrome and is categorized into three clinical types with 14 subtypes, following causative mutations in different genes and loci [1,2]. Despite these impressive statistics, Usher Syndrome has an estimated prevalence of only 0.0044% in the United States, suggesting chronic underdiagnosis in clinical settings [1]. The sensory deficits of this condition are secondary to profound bilateral sensorineural hearing loss and retinitis pigmentosa, respectively [2,3]. It is believed "far senses" [hearing and vision] are necessary to participate in the complexities of human communication [1,4]. In the absence of one, the remaining sense will compensate to preserve function; however, when both senses are compromised, the "near senses" [smell, taste, and tactility] will augment but fall short of complete compensation [1,4]. With this in mind, deaf-blindness as a combined disability is immensely debilitating and greatly limits available activities and societal participation with lasting effects in "social life, communication, access to information, orientation, and the ability to move around freely and safely [1]." In addition to hearing and visual loss, many patients with this syndrome have concomitant bilateral vestibular areflexia [1]. The vestibular system consists of two organs: the two otolith organs [utricle and saccule] and the three semicircular canals [anterior, posterior, and horizontal], which detect linear acceleration and rotational acceleration, respectively [5]. The sensations from these organs are complex and constant, yet they operate entirely subconsciously in light of the lack of a perceivable sensation [5]. A genetic condition marked by vestibular areflexia will naturally manifest as balance abnormalities in affected patients [1,3]. 

In developed nations, Usher Syndrome is primarily diagnosed in pediatric populations. This results from both legislative mandates, which vary from state to state, and subsequent access and ability to readily perform the Universal Newborn Hearing Screening (UNHS) on every newborn [6]. Initially, a patient will clinically present with some degree of perceived hearing loss, a finding usually reported by a parent or caretaker. The Early Hearing Detection and Intervention (EDHI) guidelines [7], published by the Joint Committee on Infant Hearing (JCIH), propose a timeline to understand better the clinical approach to sensorineural hearing loss [8]. In the United States, sensorineural hearing loss occurs in 0.2% to 0.4% of live births, affecting up to 40,000 children annually, two-thirds of whom experience bilateral hearing compromise [9,10]. The guidelines dictate an assignment of a medical home at birth with immediate completion of the UNHS or rescreening by one month of age [8]. If this assessment is failed, the patient should be referred for pediatric audiology assessment by 2 to 3 months of age, enrolled in their residing states' First Steps/Early Intervention Program (as allotted by the Individuals with Disabilities Education Act-Part C) [11] and referred to an otolaryngologist by 3 to 6 months of age [8]. It is also encouraged to schedule follow up with ophthalmology and genetic specialists; however, timelines for these specialties are not included in the current guidelines [7,8].

The next clinical progression in Usher Syndrome is progressive blindness due to retinitis pigmentosa. This is usually adolescent-onset and involves a progressive, bilateral, and symmetric degeneration of the retina's rod and cone functional cells [3]. Distinctive pigments are left behind as the retina degenerates, giving this condition its name [1]. Research shows the cellular basis of this condition is the result of compromised photoreceptors, retinal pigment epithelial cells, and Müller cells [12-14]. The eye's rods are affected first, leading to an inability to see in darkness, then degeneration of the cones will start at the periphery and work centrally, progressing from "tunnel vision" to complete visual compromise [13].

Research into Usher Syndrome has identified up to nine causative genes and at least 12 chromosomal loci as potential sources of disease [1,15]. Naturally, these genes correspond to a protein, most belonging to different protein classes and families [15]. USH seems to interfere exclusively with sensory cells that express actin filaments in specific segments. This may explain why the disease essentially affects the eyes and ears [1]. The actual defect lies within the structural and functional proteins of neural crest cells that compose both the inner ear and the retina [1,2]. Additionally, because the cochlea and vestibular apparatus originate from neural crest tissue, which becomes the otic vesicle, Usher Syndrome patients are more likely to present ataxic symptoms [16]. Progressive damage to the vestibular system and the more apparent inner ear damage may give another clinical indication that the patient has Usher Syndrome [2,17]. 

For a diagnosis of Usher Syndrome, both copies of the responsible gene must be deficient for the condition to arise, in line with its autosomal recessive inheritance pattern. It should be noted that digenic, bi-allelic, and polygenic forms of this condition have also been reported, in addition to dominant or nonsyndromic forms of genetic mutations [1]. Despite symptoms following the distribution and expression of different proteins in different organs, the variability in the expression of similar dysfunctions can be remarkable, even among siblings. Penetrance is considered about 100% for all types, with the most severe forms (USH1) being characterized by congenital profound sensory hearing loss, bilateral vestibular areflexia, and early onset of retinitis pigmentosa (RP) [3]. Genetic counseling is the next best step if clinical features fail to secure a diagnosis [3]. Research shows a plethora of potential causes for this condition; thus, Usher Syndrome has been divided into three clinical types: 1, 2, and 3 (USH1, USH2, USH3) identified by clinical investigation, and different subtypes correspond to different genes, identified by genetic investigation. They are clinically divided based on several criteria: the severity of hearing loss, the onset of retinitis pigmentosa, and the presence of vestibular disturbance [18,19]. However, genetic investigations are essential to confirm or reject the clinical diagnosis. It is divided into 14 subtypes based on the specific gene mutation [19]. USH1 is responsible for about one-third to one-half of cases and is considered to be the most severe of the three, being marked by non-progressive profound to severe congenital bilateral sensorineural hearing loss, near complete vestibular areflexia, and the pre-pubertal onset of retinitis pigmentosa [1,18,19].

Most patients with Usher Syndrome require a collaboration of specialties to ensure proper development. For example, without using cochlear implants (with or without previous use of hearing aids), patients will likely fail to develop speech, and delayed introduction of sign language and tactile forms of learning [braille] can compromise the patient's ability to interact as they age. An absence of vestibular compensation therapies can promote life-long balance complications [3]. A close medical relationship will be needed throughout the patient's life, and requirements will only increase as the patient's condition develops [1,3].

## Case presentation

A healthy newborn female, born via spontaneous vaginal delivery to a G2P1001 female with 1-minute and 5-minute APGAR scores of 8 and 9, respectively, underwent all necessary neonatal care and prophylaxis according to protocol. Included in this care was the UNHS, which the patient failed bilaterally. No further postpartum complications were encountered, and they were discharged at 48 hours of age. The patient's parents were advised to follow up with a pediatrician to reevaluate the patient's hearing. 

At four days of age, the patient was taken to the pediatrician, where the parents raised additional concerns. They reported that the infant did not appear to respond to any sound, using the barking of the family dogs as an example. The patient's hearing was then reevaluated at one month of age, which resulted in another bilateral failure. With no gross abnormalities noted on the otoscopic examination, it was determined that the infant would need further evaluation by a pediatric otolaryngologist (ENT) to determine the potential cause and to categorize the severity of the infant's hearing loss, upon referral to ENT, another otoscope exam determined patent external auditory canals with a normal appearing tympanic membrane. A high-frequency tympanogram determined there were no middle ear abnormalities. Auditory Brainstem Response (ABR) testing confirmed bilateral severe-to-profound sensorineural hearing loss. 

To prevent any delays in milestones, the patient was scheduled to receive cochlear implants, and speech therapy was recommended. With no other complaints or obvious causes, a geneticist was consulted to determine if an underlying genetic abnormality would explain her sensorineural hearing loss. While awaiting genetic testing, the patient was found to be mildly hypotonic at her four-month checkup, failing to meet major motor milestones such as rolling from prone. At six months, parents reported the infant could not roll and had difficulty sitting up with support. At nine months, the patient showed developmental milestones more consistent with a 5-6-month-old. Genetic testing revealed that the child carried two variants of uncertain significance in the Unconventional Myosin VII-A (MYO7A) gene, consistent with Usher Syndrome. 

These MYO7A gene variants were confirmed to be on opposite chromosomes, and the laboratory upgraded one of the variants from "uncertain significance" to "likely pathogenic." Although one variant of the patient's MYO7A gene remains of "uncertain significance," the variant has been identified in other patients with Usher Syndrome, and the geneticist anticipates this variant to be upgraded in the future. The patient's diagnosis remains a clinical diagnosis based on the pathogenic variant of the MYO7A gene and her clinical presentation.

With the diagnosis of Usher Syndrome being confirmed, the pediatrician referred the patient to a pediatric specialist to better assess and manage the deficits associated with this condition. An ears, nose, and throat specialist continued management of the cochlear implants. An ophthalmologist was consulted for the management of ocular deficits. A neurology referral was given to assess the vestibular deficits, most likely delaying the patient's motor coordination. Finally, physical and speech therapy were consulted for motor and speech deficits. Genetic counseling was also provided for the parents. Geneticists gave them the information moving forward if they were to have another child and the probability of that child being diagnosed with Usher Syndrome. Together with the specialist, the pediatrician has started the patient on a treatment plan to ensure the quality of life is prioritized. 

## Discussion

Genetic testing should be prioritized if a pediatrician or general practitioner suspects Usher Syndrome. Table 1 shows several known genetic causes for this condition, furthering the importance of early and accurate genetic screening.

**Table 1:** The genetic variations associated with each clinical type of Usher Syndrome

Usher syndrome type:	Usher syndrome subtypes:	Associated gene mutation:
Type 1	B	MYO7A
	C	USH1C
	D	CDH23
	F	PCDH15
	G	USH1G
	J	CIB2
Type 2	A	USH2A
	C	ADGRV1
	D	WHRN
Type 3	A	CLRN1
	B	HARS

Early genetic testing was initiated in this case, and the MYO7A gene mutation was identified, leading to the diagnosis of USH1B, the most severe form of Usher Syndrome. With these patients developing severe hearing loss, equilibrium disorders, and early onset retinitis pigmentosa, physicians must have a broad differential that includes Usher Syndrome when a newborn patient fails a hearing screening. Due to the varying degree of clinical presentations and the rarity of Usher Syndrome, it is suspected that many mild forms have gone undiagnosed or been incorrectly diagnosed [1]. The diagnosis is important because of the delay in speech and language development due to hearing loss and the potential walking delays and ataxic difficulties that may result from vestibular abnormalities [2]. Physicians should be suspected of Usher Syndrome if an infant presents with sensorineural hearing loss and failure to walk, and no other diagnoses have been made. These are good clinical indicators a child could have Usher Syndrome, and genetic counseling should be sought for further investigation [2]. Physician intervention is a priority for these patients to ensure the child gets the necessary medical interventions that are required to ensure developmental milestones are met. Hearing aids may suffice, but cochlear implants may be required as definitive treatment. Neurology should also be consulted for vestibular dysfunction, and an ophthalmologist to continue monitoring for retinal degeneration, cataracts, and/or cystoid macular edema [18]. Physical therapy should be readily available for these patients and should be tailored to the patient's degree of vestibular dysfunction. Speech therapy should be initiated at a young age to mitigate developmental deficits.

## Conclusions

In total, Usher Syndrome is believed to be the most common heritable form of combined hearing and visual loss across populations. This case study follows a newborn female who failed her UNHS bilaterally in the neonatal period and at her 1-month follow-up appointment. Following a genetic workup, this patient was ultimately found to have genetic abnormalities associated with the most severe form of Usher Syndrome, USH1B. Although her diagnosis remains clinical at this time, this is believed to change secondary to the results of her genetic analysis. Ultimately, pediatric and general practitioners must keep Usher Syndrome on their differential diagnosis when a young patient presents with sensorineural hearing loss. These patients will require ample medical resources and specialty visits as their condition continues to progress, and the earlier these deficits are detected, the better the overall prognosis for the patient at hand.
